# A phase 1 study of crenigacestat (LY3039478), the Notch inhibitor, in Japanese patients with advanced solid tumors

**DOI:** 10.1007/s10637-020-01001-5

**Published:** 2020-09-16

**Authors:** Toshihiko Doi, Masaomi Tajimi, Joji Mori, Hiroya Asou, Koichi Inoue, Karim A. Benhadji, Yoichi Naito

**Affiliations:** 1 National Cancer Center Hospital East, 5-1, Kashiwanoha 6-chome, Kashiwa-shi, Chiba 277-8577 Japan; 2 Eli Lilly Japan K.K, Kobe, Japan; 3 Eli Lilly and Company, New York, NY USA

**Keywords:** Crenigacestat, Japanese, LY3039478, Notch pathway, Phase 1, Solid tumor

## Abstract

*Background* This phase 1, single-center, nonrandomized, single-arm, open-label, dose-escalation study, evaluated the tolerability of crenigacestat, a γ-secretase inhibitor as an oral Notch inhibitor in Japanese patients with advanced solid tumors. *Methods* The study consisted of 2 dose levels of crenigacestat (25 mg and 50 mg), administered orally 3 times per week (TIW) over a 28-day cycle until disease progression, development of unacceptable toxicity, or any other discontinuation criteria were met. The primary objective was to evaluate the tolerability and determine the recommended dose of crenigacestat for Japanese patients. Secondary objectives were to characterize the safety and toxicity, the pharmacokinetic parameters, and to document any antitumor activity of crenigacestat. *Results* Eleven Japanese patients with advanced solid tumors were enrolled; 4 patients (median age of 64 years) received 25 mg of crenigacestat, and 7 patients (median age of 72 years) received 50 mg of crenigacestat. Median treatment duration was 8 weeks in the 25-mg treatment arm and 4 weeks in the 50-mg treatment arm. There were no dose-limiting toxicities or dose-limiting equivalent toxicities observed. None of the patients had a complete or partial response to the treatment. One patient (14.3%) with a desmoid tumor in the 50-mg treatment arm showed tumor size shrinkage of 22.4% and had stable disease for 22.5 months. Frequent (>14%) treatment-related-adverse events in both treatment arms included diarrhea, malaise, and vomiting. *Conclusions* Crenigacestat was tolerated in Japanese patients but with limited clinical activity. The recommended crenigacestat dose in Japanese patients is 50 mg TIW.

**Trial registration:** NCT02836600 (ClinicalTrials.gov) registered on July 19, 2016.

## Introduction

The Notch signaling pathway is evolutionarily conserved in mammals and plays an important role in cell development and differentiation [1]. In mammals, there are four isoforms of Notch receptors (Notch-1, Notch-2, Notch-3, and Notch-4) [2] and five Notch ligands (Dll-1, Dll-3, Dll-4, Jagged-1, and Jagged-2) [3], which are vital in mediating communication between adjacent cells expressing these receptors and ligands [4].

A substantial body of evidence suggests that Notch signaling plays important oncogenic roles in several types of cancer [4]. The oncogenic functions of Notch signaling include the inhibition of apoptosis and the promotion of cell proliferation [5]. Deregulated Notch signaling due to mutation or overexpression of ligands and/or receptors is implicated in a number of malignancies, including lymphoid leukemia, melanoma, glioblastoma, and cancers of the breast, ovary, lung, pancreas, colon, head and neck, cervix, and kidney [6–8]. Thus, targeting components of the Notch signaling pathway may be a relevant option for cancer treatment.

Crenigacestat is a potent small molecule inhibitor of Notch cleavage that prevents the release of the Notch intracellular domain by inhibiting proteolytic activity of γ-secretase complex, and thereby decreasing Notch signaling and its downstream biologic effects. A phase 1, nonrandomized, open-label, multicenter trial that evaluated the safety and antitumor activity of crenigacestat in non-Japanese patients with advanced or metastatic cancers recommended a phase 2 dose of crenigacestat monotherapy at 50 mg administered 3 times per week (TIW) during a 28-day cycle [9]. In the confirmatory, expansion trial of crenigacestat in patients with adenoid cystic carcinoma, it was demonstrated that crenigacestat has a manageable safety profile and a clinical pharmacodynamic effect on Notch-targeted genes. At the recommended phase 2 dose, the most frequent toxicities included diarrhea, nausea, and vomiting [9]. Clinical activity (tumor necrosis, metabolic response, or tumor shrinkage) was observed in patients with breast cancer, leiomyosarcoma, and adenoid cystic carcinoma [9]. Crenigacestat was further explored in patients with adenoid cystic carcinoma and sarcoma [10, 11].

In this phase 1, single-center, nonrandomized, single-arm, open-label, dose-escalation study, we evaluated the tolerability of crenigacestat up to the global recommended dose in Japanese patients with advanced solid tumors.

## Methods

### Study design

I6F-JE-JJCC was a phase 1, single- center, nonrandomized, single-arm, open-label, dose-escalation study of crenigacestat (LY3039478) in Japanese patients with advanced solid tumors (NCT02836600). The primary objective was to evaluate the tolerability of crenigacestat up to the global recommended dose in Japanese patients with advanced solid tumors. Secondary objectives were to characterize the safety and toxicity profile, to evaluate the pharmacokinetic (PK) parameters, and to document any antitumor activity of crenigacestat. This study was conducted in compliance with the Declaration of Helsinki, Council for International Organizations of Medical Sciences International Ethical Guidelines, International Conference on Harmonization Guidelines for Good Clinical Practice, and applicable local regulations. The ethics committees at the participating center approved the protocol, and all patients provided written informed consent before study entry.

### Patients

Eligible patients were Japanese (≥20 years of age) with histological or cytological evidence of advanced and/or metastatic solid tumor for whom standard therapies failed or would not be appropriate. Patients had measurable and/or nonmeasurable disease as defined by the Response Evaluation Criteria in Solid Tumors version 1.1 (RECIST v1.1) [12], the Eastern Cooperative Oncology group (ECOG) performance status of ≤1, and adequate organ function. Patients discontinued all previous therapies for cancer (including chemotherapy, radiotherapy, immunotherapy, and investigational therapy) for at least 21 days for myelosuppressive agents or 14 days for nonmyelosuppressive agents prior to receiving study drug. Patients were excluded if they had received treatment with any study drug that had not received regulatory approval for any indication within 14 or 21 days of the initial dose of study drug for a nonmyelosuppressive or myelosuppressive agent, respectively. Patients were not permitted in the study if they had a serious preexisting medical condition, received prior treatment with a Notch inhibitor, had persistent bleeding, or had undergone major surgery within 28 days prior to the first dose.

### Study treatment and dose escalation

The study consisted of 2 dose levels of crenigacestat (25 mg and 50 mg), administered orally TIW prior to a meal. A cycle was defined as 28 days. Patients were admitted to the investigational site for 4 weeks through the entire first cycle but were discharged and managed on an outpatient basis on or after day 15 upon investigator discretion. The planned duration of treatment was not fixed. Treatment continued until disease progression, development of unacceptable toxicity, or any other discontinuation criteria were met.

Data were evaluated on an ongoing basis until the tolerability of crenigacestat for 50 mg was confirmed. Safety data, in particular adverse events (AEs), were the primary criteria for dose escalation. Dose escalation was driven for each treatment combination using the 3 + 3 method. Transition of dose level (from 25 to 50 mg) proceeded if the frequency of dose-limiting toxicity (DLT) observed in cycle 1 was <33% of patients in the first dose level (25 mg). For DLTs, an evaluable patient was defined as a patient who received ≥75% of the planned dose in cycle 1 or a patient who experienced DLT in cycle 1.

### Safety assessment

Adverse events were collected throughout the study and coded using Medical Dictionary for Regulatory Activities (MedDRA) terms (Version 22.0). Dose-limiting toxicities were defined as AEs during cycle 1 that were related to crenigacestat and fulfilled any one of the following criteria using the National Cancer Institute Common Terminology Criteria for Adverse Events (CTCAE) v 4.0: CTCAE grade ≥ 3 non-hematological toxicity (exceptions made for nausea, vomiting, or constipation that lasts <72 h and can be controlled with treatment; transient grade 3 elevations of alanine aminotransferase and/or aspartate aminotransferase), CTCAE grade 4 hematological toxicity of >5 days duration, any febrile neutropenia, grade 3 thrombocytopenia with bleeding, or grade 4 thrombocytopenia, and other significant toxicity deemed to be dose limiting by the investigator. Dose-limiting equivalent toxicities (DLETs) were defined as an AE occurring in any cycle (other than cycle 1) that meets the criteria for a DLT if it had occurred during cycle 1.

### Efficacy assessment

Tumor response, including overall response rate, was measured using RECIST v1.1 [12] or the Response Assessment in Neuro-Oncology criteria for glioblastoma [13]. Patient’s full extent of disease was also assessed via evaluation of performance status (ECOG). Where applicable, tumor measurements were performed by positron emission tomography response criteria of the European Organization for Research and Treatment of Cancer [14], as well as evaluation of tumor markers. To confirm objective responses, all lesions were radiologically assessed and the same radiologic method used at baseline and for the initial response determination was repeated at least 4 weeks following the initial observation of an objective response.

### Pharmacokinetics

All patients who received at least 1 dose of the study drug, and had sufficient samples collected, were included in the PK analysis. Samples were collected for PK analysis up to 24 h following the first dose of crenigacestat. Plasma concentrations of crenigacestat were quantified using a validated liquid chromatography-tandem mass spectrometry (LC-MS/MS) assay. The PK parameters for crenigacestat were calculated by standard noncompartmental methods of analysis. The primary PK parameters for analysis were maximum plasma concentration (C_max_) and area under the plasma concentration-time curve (AUC) from time zero to infinity or over 1 dosing interval at steady state of crenigacestat.

### Statistical analyses

Data from all patients who received at least 1 dose of crenigacestat treatment were included in the summaries of safety and efficacy. The analyses for this study were descriptive, except for possible exploratory analysis, as deemed appropriate. The sample size was determined by the study design, rather than a statistical power calculation. Continuous variables were presented using the mean, standard deviation (SD), median, minimum, maximum, and number of patients with an observation. For categorical variables, the population size, number of events, number of subjects with events, and percentage of subjects with events were reported.

## Results

### Patient characteristics

The planned study population was 3 and 6 patients for the 25 mg and 50 mg treatment groups, respectively. An additional patient was evaluated in the 25-mg treatment group due to DLT evaluation being inconclusive in 1 patient. Furthermore, 1 patient in the 50-mg treatment group was replaced during cycle 1 due to progressive disease, with DLT unable to be evaluated. Overall, 11 Japanese patients were enrolled in the study: 4 patients with a median age of 64 years (min-max 58–71) received 25 mg of crenigacestat, and 7 patients with a median age of 72 years (min-max 36–80) received 50 mg of crenigacestat.

Patients in the 25-mg crenigacestat treatment arm had colon cancer (*n* = 3) and gastric cancer (*n* = 1). Patients in the 50-mg crenigacestat treatment arm had colon cancer (*n* = 2), gastric cancer (n = 1), malignant melanoma (n = 2), prostate cancer (n = 1), and desmoid tumor (n = 1). All patients had a histopathological diagnosis of which 3 patients (75.0%) in the 25-mg treatment arm and 2 patients (28.6%) in the 50-mg treatment arm had a well-differentiated (low-grade) diagnosis. With the exception of 1 patient (14.3%) in the 50-mg treatment arm with an ECOG performance status of 1 at baseline, all the other patients had a score of 0 at baseline. Patient demographics and baseline disease characteristics are outlined in Table 1.

**Table 1:** Patient and disease characteristics of Japanese patients with solid tumors

Characteristics	Crenigacestat 25 mg (n = 4)	Crenigacestat 50 mg (*n* = 7)
Gender		
Male	2 (50.0)	5 (71.4)
Female	2 (50.0)	2 (28.6)
Age, years, median (range)	64 (58–71)	72 (36–80)
ECOG		
0	4 (100.0)	6 (85.7)
1	0	1 (14.3)
Tumor type		
Colon cancer	3 (75.0)	2 (28.6)
Adenocarcinoma gastric	1 (25.0)	1 (14.3)
Malignant melanoma	0	2 (28.6)
Prostate cancer	0	1 (14.3)
Desmoid tumor	0	1 (14.3)
Prior Systemic Treatment		
1	0	0
≥2	4 (100.0)	7 (100.0)

Data are n (%) unless otherwise indicated

*Abbreviations*: *ECOG* Eastern Cooperative Oncology Group, *n* number of patients

#### Safety

The DLT analyses set consisted of 4 patients in the 25-mg treatment arm and 6 patients in the 50-mg treatment arm. DLT evaluation for 1 patient in the 25-mg treatment group was inconclusive as it was unclear whether the AE of increased hepatic enzyme (grade 3) was due to study treatment. One patient in the 50-mg treatment group discontinued on day 8 due to progressive disease, and hence was not included for DLT evaluation.. There were no DLTs or DLETs observed in either treatment arm. All 4 patients (100%) in the 25-mg treatment arm and 6 patients (85.7%) in the 50-mg treatment arm exhibited at least 1 treatment emergent adverse event (TEAE) each.

The system organ class (SOC) with the most frequently reported TEAEs was the gastrointestinal (GI) SOC; *n* = 2 (50.0%) in the 25-mg treatment arm and *n* = 4 (57.1%) in the 50-mg treatment arm. The most frequently reported TEAE by preferred term was diarrhea experienced by 1 patient (25.0%) in the 25-mg treatment arm and 3 patients (42.9%) in the 50-mg treatment arm. The majority of diarrhea cases were considered due to study treatment (*n* = 1 in 25-mg treatment arm, and *n* = 2 in 50-mg treatment arm); however, 1 patient in the 50-mg treatment arm had 2 TEAEs of diarrhea, and both events were considered not related to study treatment. All cases of diarrhea were of low grade in severity (grade 1) and were resolved. There were no TEAEs of grade 4 or 5 severity reported. The most frequently reported TEAEs are outlined in Table 2.

**Table 2:** Most frequent TEAEs related to study treatment by maximum CTCAE grade categories

Adverse events	Crenigacestat 25 mg (n = 4)			Crenigacestat 50 mg (n = 7)		
	Grade 1	Grade 2	Grade 3	Grade 1	Grade 2	Grade 3
Gastrointestinal disorders	2 (50.0)	0	0	1 (14.3)	2 (28.6)	0
Diarrhoea	1 (25.0)	0	0	2 (28.6)	0	0
Vomiting	1 (25.0)	0	0	1 (14.3)	0	0
Nausea	0	0	0	1 (14.3)	2 (28.6)	0
Malaise	1 (25.0)	0	0	1 (14.3)	0	0
Metabolism and nutrition disorders	0	0	0	0	2 (28.6)	2 (28.6)
Decreased appetite	0	0	0	1 (14.3)	2 (28.6)	0
Hypophosphataemia	0	0	0	0	0	2 (28.6)
Hepatic enzyme increased	0	0	1 (25.0)	0	0	0
Eye disorders	1 (25.0)	0	0	0	0	0
Visual impairment	1 (25.0)	0	0	0	0	0
Skin and subcutaneous tissue disorders	0	0	0	2 (28.6)	0	0
Rash	0	0	0	1 (14.3)	0	0
Alopecia	0	0	0	1 (14.3)	0	0
Hair color changes	0	0	0	1 (14.3)	0	0

Data reported as n (%). Adverse events were coded using MedDRA terms (Version 22.0)

*Abbreviations*: *CTCAE* Common Terminology Criteria for Adverse Events, *MedDRA* Medical Dictionary for Regulatory Activites, *n* number of patients

There were no patients who reported any serious adverse events (SAEs), discontinued study treatment due to an adverse event (AEs) or SAE, or died due to an AE on study treatment or within 30 days of discontinuation from study treatment. However, 1 patient died within 30 days of discontinuation from study treatment due to study disease. There were no patients who showed any clinically significant changes in any safety parameters, including vital signs and laboratory parameters.

One patient (25.0%) in the 25-mg treatment arm and 5 patients (71.4%) in the 50-mg treatment arm experienced TEAEs that led to dose omissions. The reason for dose omission related to the study treatment in the 25-mg treatment arm was increased hepatic enzymes (grade 3, *n* = 1). For the 50-mg treatment arm, dose omissions considered related to the study treatment were due to decreased appetite (grade 1, *n* = 2) and nausea (grade 2, n = 1), while urinary tract infection (grade 2, n = 1) and supraventricular tachycardia (grade 2, n = 1) were not considered related to the study treatment.

#### Efficacy

**Fig. 1:**
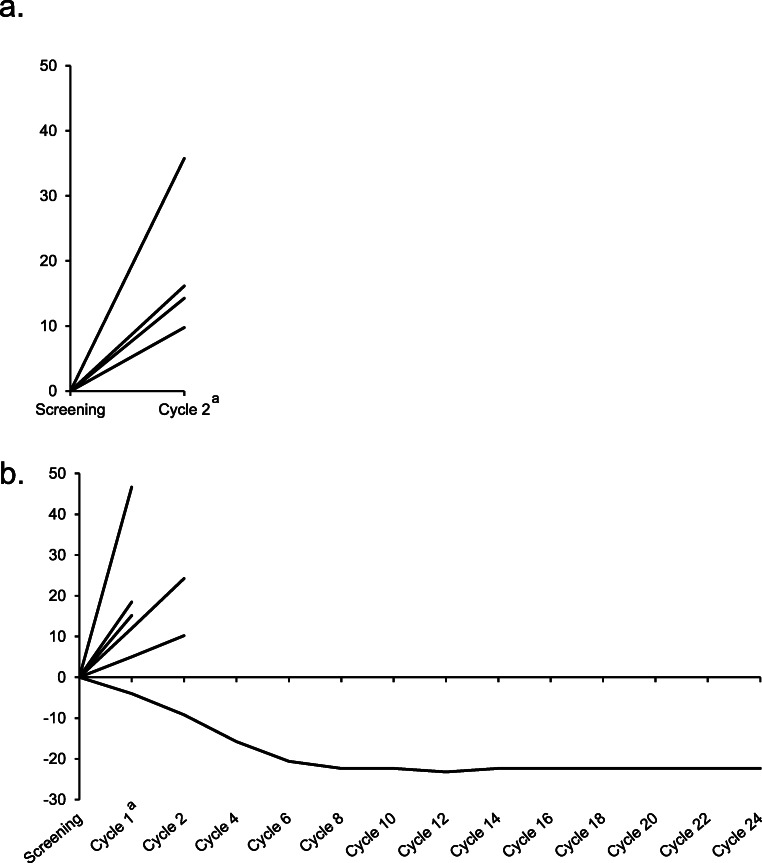
Change from baseline in tumor size in Japanese patients with solid tumors. Percent change from baseline in Japanese patients with solid tumors receiving a. 25 mg of crenigacestat (*n* = 4) or b. 50 mg of crenigacestat (*n* = 6). Each line represents individual patients treated with crenigacestat. One patient in the 50-mg treatment group discontinued on day 8 and hence data for this patient is not shown. ^a^Cycle duration was 28 days

The median duration of therapy in the 25-mg treatment arm was 8.0 weeks (min-max 8.0–8.0) and in the 50-mg treatment arm was 4.0 weeks (min-max 4.0–100.0). None of the patients had a complete or partial response to the treatment. One heavily pretreated patient with a desmoid tumor in the 50-mg treatment arm showed tumor size shrinkage of 22.37% during the first 8 cycles of the study treatment and had stable disease for the next 16 cycles (22.47 months). The remainder of the patients in both treatment arms had objective progressive disease. A summary of best overall response is outline in Table 3. The change from baseline in tumor size is shown in Fig. 1.

**Table 3:** Best overall response

	Crenigacestat 25 mg (n = 4)	Crenigacestat 50 mg (n = 7)
Best overall response		
Complete response (CR)	0	0
Partial response (PR)	0	0
Stable disease (SD)	0	1 (14.3)
Progressive disease	4 (100.0)	6 (85.7)
Disease control rate (CR/PR/SD)	0	1 (14.3)

Data reported as n (%)

*Abbreviations*: *n* number of patients

### Pharmacokinetics

**Fig. 2:**
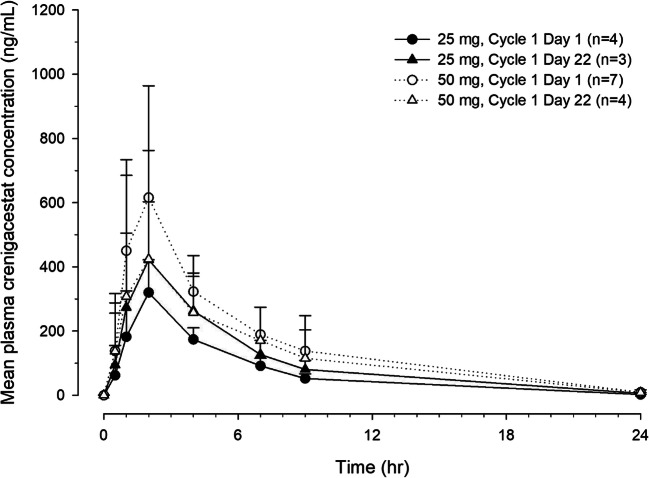
Plasma crenigacestat concentration-time profiles in Japanese patients with solid tumors. Mean (±SD) plasma concentration-time profiles on days 1 and 22 during the first cycle following thrice weekly oral doses of 25 mg or 50 mg of crenigacestat in Japanese patients with solid tumors. Abbreviations: hr, hour; n, number of patients; SD, standard deviation

Pharmacokinetics was assessed in all 11 patients. The crenigacestat PK profile was characterized by rapid absorption and elimination. Median time to maximum plasma concentration (t_max_) occurred approximately 2 h postdose for each dose level. The mean t_1/2_ was approximately 4 h, suggesting that no accumulation of crenigacestat occurs with dosing TIW. Pharmacokinetic exposures appeared to increase with dose on day 1, but this was not apparent on day 22. Note that interpretation is limited due to the small numbers of patients per dose level. Pharmacokinetic data is summarized in Table 4, and the plasma crenigacestat concentration-time profile is shown in Fig. 2.

**Table 4:** Pharmacokinetic parameters in Japanese patients with solid tumors following TIW oral doses of crenigacestat on days 1 and 22^a^

	Day 1 (Dose 1)		Day 22 (Dose 10)	
	Crenigacestat 25 mg	Crenigacestat 50 mg	Crenigacestat 25 mg	Crenigacestat 50 mg
N^b^	4/4	7/6	3/3	4/3
t_max,_ median (range), h	1.92 (1.00–1.93)	1.93 (1.08–8.03)	1.88 (0.95–1.92)	1.94 (0.88–5.85)
C_max_, ng/mL	324 (34)	670 (39)	429 (69)	416 (70)
AUC_0–48_, ng*h/mL	1480 (20)	3080 (38)	2070 (54)	2090 (76)
AUC_0-∞_, ng*h/mL	1480 (20)	3080 (38)	–	–
t½, geometric mean (range), h	3.64 (3.36–3.82)	3.89 (2.94–4.84)	4.19 (3.66–4.71)	3.67 (3.40–4.14)

*Abbreviations*: *AUC*_*0–48*_ area under the plasma drug concentration versus time curve from time 0 to 48 h, *AUC*_*0-∞*_ area under the plasma drug concentration versus time curve from time zero to infinity, *C*_*max*_ maximum observed concentration, *%CV* percent coefficient of variation, *N* number of patients, *PK* pharmacokinetic; t_½,_ elimination half-life; TIW, 3 times per week; t_max,_ time to reach C_max_

^a^Data presented as geometric mean (%CV) unless otherwise indicated

^b^Two N values reported. First N value is for C_max_ and t_max_, while the second N value is for remaining parameters that are dependent on terminal phase of PK profile. Parameters dependent on terminal phase only reported when 24-h time point result is available and when ([AUC0-∞-AUClast]/AUC0-∞)*100 ≤ 15%

## Discussion

This report describes the first clinical study of crenigacestat in Japanese patients with solid tumors. Results of this phase 1, single-center, nonrandomized, single-arm, open-label, dose-escalation study indicate that crenigacestat is tolerated at both the 25 mg and 50 mg doses in Japanese patients, with 50 mg TIW confirmed as the recommended phase 2 dose in this population [9].

The duration of treatment was relatively short for both treatment arms (median treatment duration of 8 weeks and 4 weeks for 25-mg and 50-mg crenigacestat treatment arms, respectively). The majority of TEAEs were mild or moderate in intensity, and no TEAEs of grade 4 or 5 in severity were reported. Gastrointestinal disorders and toxicities (including diarrhea and vomiting) were among the most commonly reported TEAEs related to study treatment. These GI toxicities were manageable, and none led to study discontinuation. This is consistent with previously reported dose escalation and other dose expansion cohorts of crenigacestat [9–11]. The PK profile of crenigacestat observed in this study was generally consistent with PK data reported in global phase 1 studies [9–11].

There were no partial or complete responses to treatment in Japanese patients at either the 25 mg or 50 mg doses of crenigacestat, and the majority of patients exhibited progressive disease. Similar to the current report, a recent phase 1 study of crenigacestat demonstrated manageable toxicity and limited clinical activity, with no confirmed responses, in patients with adenoid cystic carcinoma [10]. However, we observed one female patient (age 36 years) in the 50-mg treatment arm with stable disease for 22.5 months and tumor shrinkage of 22.4%. This patient had a desmoid tumor (aggressive fibromatosis), which is clinically important as there are currently no established or evidence-based treatment options available for this disease. This patient was enrolled in the current study because the mode of action of crenigacestat is predicted to be effective in the treatment of desmoid tumors. Desmoid tumors are often locally aggressive and are driven by aberrations within the WNT/β-catenin pathway [15, 16]. Along with overexpression of β-catenin, desmoid tumors have been shown to highly express *NOTCH1* and its downstream transcription factor *HES1* [17].

Interestingly, the Notch pathway is thought to be a therapeutic target for these tumors [15, 16]. Antitumor activity by single agents targeting the Notch signaling pathway, such as a γ-secretase inhibitor and a monoclonal antibody targeting Notch 2/3 receptors, have been observed in early phase clinical trials of sarcoma or desmoid tumors [11, 18–23]. Hence, there is potential for the use of crenigacestat and other inhibitors of the Notch signaling pathway in the treatment of sarcoma.

Limitations of this study were the small sample size and the nonrandomized, single-arm study design, although this is typical of dose-escalation studies. Overall, crenigacestat monotherapy and other notch inhibitors have shown modest or limited activity in early phase trials of solid tumors. Exploration of potential predictive biomarkers to identify patients most likely to benefit from Notch monotherapy is warranted. Trials focusing on non-solid tumors, such multiple myeloma, are being explored due to the modulation of BCMA by γ-secretase inhibitors. Future evaluation of crenigacestat in Japanese patients may be warranted.

## Conclusions

Overall, crenigacestat was tolerated at both the 25 mg and 50 mg doses in Japanese patients with solid tumors. However, no clinical activity of crenigacestat was observed at the recommended dose in this patient population, and there was no confirmed objective response during the observation period of this study.

## Data Availability

Eli Lilly and Company provides access to all individual participant data collected during the trial, after anonymization, with the exception of pharmacokinetic or genetic data. Data are available to request 6 months after the indication studied has been approved in the US and EU and after primary publication acceptance, whichever is later. No expiration date of data requests is currently set once data are made available. Access is provided after a proposal has been approved by an independent review committee identified for this purpose and after receipt of a signed data sharing agreement. Data and documents, including the study protocol, statistical analysis plan, clinical study report, and blank or annotated case report forms, will be provided in a secure data-sharing environment. For details on submitting a request, see the instructions provided at www.vivli.org
